# Kyste hydatique mammaire primitive

**DOI:** 10.11604/pamj.2015.20.385.6318

**Published:** 2015-04-17

**Authors:** Houssine Boufettal, Naïma Samouh

**Affiliations:** 1 Service de Gynécologie –Obstetrique, Hopital Ibn Rochd, Université Hassan 2, Faculté de Médecine et de Pharmacie, Casablanca, Maroc

**Keywords:** Kyste, hydatique, échinococcose, sein, primitive, cyst, hydatid, echinococcosis, breast, primitive

## Abstract

La localisation mammaire du kyste hydatique est exceptionnelle. De ce fait, le diagnostic est difficile avant l'examen anatomopathologique. Nous rapportons une observation d'un cas de kyste hydatique du sein chez une femme de 32 ans, qui consultait pour un nodule du sein, dont l'imagerie montrait une lésion en rétro-aréolaire du sein gauche, homogène, ovalaire et de contours réguliers. L'examen anatomopathologique objectivait un kyste hydatique à localisation mammaire. Les suites opératoires étaient simples. L'hydatidose est une maladie ubiquitaire, pouvant atteindre tous les organes. Le diagnostic peut être évoqué devant une masse kystique du sein avec des aspects très évocateurs à l'imagerie. La confirmation du diagnostic n'est confirmée qu'après une cytoponction ou une chirurgie d'exérèse qui réalise le traitement de cette pathologique. La négativité du bilan d'extension hydatique permet de retenir une localisation primitive de l’échinococcose.

## Introduction

La localisation mammaire du kyste hydatique est exceptionnelle [1–3]. Cependant, et vu que l'hydatidose est une maladie ubiquitaire, le sein peut être atteint comme tous les autres organes [4]. La localisation mammaire du kyste hydatique est rare, caractérisée par son évolution lente et par l'efficacité du traitement chirurgical [2]. Le diagnostic de cette affection est habituellement aisé grâce au couple mammographie-échographie [3]. Par la rareté de cette localisation, la certitude ne peut être obtenue qu’à l'examen macroscopique de la lésion [4]. Nous rapportons une observation d'un cas de kyste hydatique du sein. A travers laquelle, les auteurs montreront les aspects diagnostiques et thérapeutiques de cette, rare, localisation.

## Patient et observation

**Figure 1:**
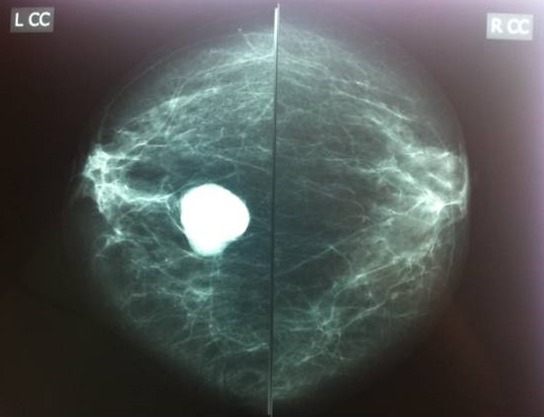
Cliché mammographique de face montrant une opacité centrale, bien limitée et homogène du sein gauche

**Figure 2:**
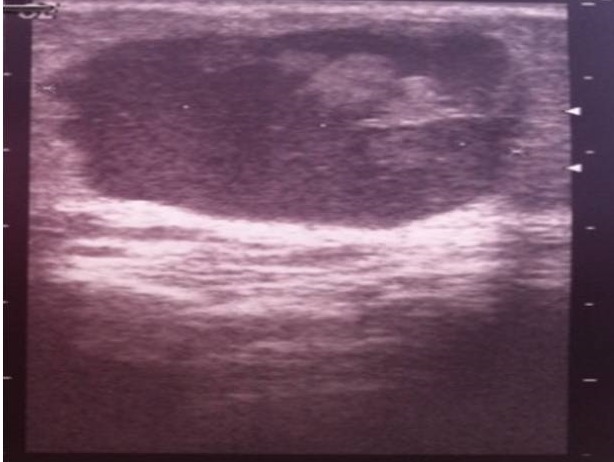
Sur le complément échographique, la lésion est liquidienne, bien limitée

**Figure 3:**
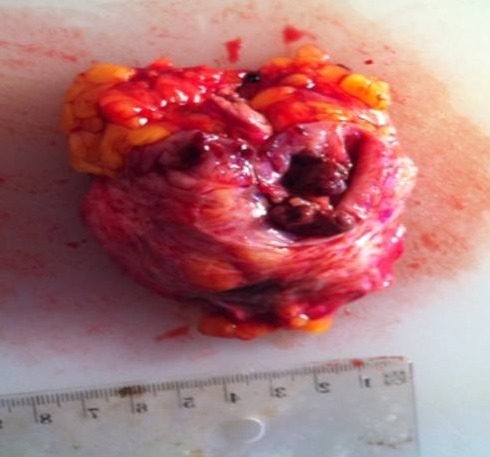
Aspect macroscopique de la pièce opératoire d'exérèse chirurgicale

Une patiente âgée de 32 ans, originaire d'un milieu rural, sans antécédents personnels ou familiaux. Elle présentait depuis deux années une tuméfaction du sein gauche, qui augmentait progressivement de volume, associée à une mastodynie. L'examen retrouvait un nodule rétro-aréolaire du sein gauche, unique, mesurant trois centimètres de grand axe, rénitent, mobile, indolore, non fixé au plan superficiel, ni au plan profond. La mammographie montrait une opacité homogène, ovalaire de contours réguliers, qui siégeait en rétro-aréolaire du sein gauche, sans calcifications (Figure 1). L’échographie mammaire montrait une formation de 34 mm, hypoéchogène hétérogène (Figure 2). La radiographie de thorax et l’échographie abdominale étaient normales. Une exérèse chirurgicale emportant le kyste était réalisée, sans effraction de celui-ci. L'examen anatomopathologique de la pièce de tumorectomie objectivait un kyste hydatique à localisation mammaire (Figure 3). Aucun traitement spécifique n’était administré. Les suites opératoires étaient simples. Avec un recul de quatre ans, aucune récidive n’était notée.

## Discussion

L'hydatidose ou échinococcose est une helminthiase provoquée par le développement chez l'homme de la forme larvaire d'un tænia dénommé Echinococcus granulosus. Cette infection parasitaire sévit de façon endémique dans de nombreux pays du bassin méditerranéen, où elle pose un problème de santé publique [1]. L'infestation humaine est accidentelle. Elle se fait uniquement par voie digestive par ingestion d'œufs de tænia échinocoque. Ceux-ci libèrent dans l'estomac l'embryon qui franchit la barrière intestinale et emprunte le système porte qui l'emporte vers le foie, premier barrage. Il peut, par la suite, gagner le système cave, le c'ur droit et le poumon, second barrage [2]. Quand ces deux filtres sont dépassés, les parasites gagnent la circulation systémique et peuvent se greffer dans les différents territoires de l'organisme, subissant une transformation kystique à l'origine du kyste hydatique [3, 4]. La localisation mammaire de l'hydatidose est exceptionnelle. Elle représente moins de 0,2% de l'ensemble des localisations hydatiques [5–8]. De ce fait, il est toujours difficile d'en poser le diagnostic en préopératoire, même dans les pays d'endémie. Le kyste hydatique est classiquement indolore, sauf dans les circonstances rares d'une tumeur volumineuse ou surinfectée [8]. Sa consistance est habituellement ferme ou rénitente faisant évoquer surtout une pathologie bénigne (adénofibrome ou kyste). Il s'agit en général d'une tumeur unifocale, rarement multifocale sans prédilection pour un sein [1], comme c'est le ca de notre patiente. L’évolution du kyste est lente, sur plusieurs années. Dans notre cas, elle était de deux années. L’échographie, examen facile, anodin, permet de visualiser directement l'image du kyste. Les aspects possibles varient selon l’évolution chronologique du kyste. Il s'agit d'une image liquidienne pure, une image liquidienne avec décollement de membranes, un kyste multi-vésiculaire, un kyste d’échostructure hétérogène ou un kyste calcifié [2]. La mammographie montre, généralement, l'aspect d'une opacité dense arrondie, bien limitée ou non sans calcifications. Cette dernière peut être bien circonscrite par un liseré de calcifications ou calcifiée dans sa quasi-totalité. Rarement, elle peut y avoir des macro-calcifications éparses et atypiques [3]. Dans notre cas, la mammographie montrait une opacité homogène, ovalaire de contours réguliers, sans calcifications et l’échographie mammaire montrait une formation de 34mm, hypoéchogène hétérogène. L'imagerie par résonnance magnétique (IRM), peut objectiver une formation en hyposignal T1, hypersignal T2 qui se réhausse de façon annulaire après injection de produit de contraste. Le recours à l'IRM peut s'avérer nécessaire pour différencier les kystes, des tumeurs malignes [4]. La cytoponction à l'aiguille fine, ramène un liquide «eau de roche» pathognomonique de l'hydatidose mammaire [1]. Si elle est faite, elle doit être, toujours, complétée par une exérèse chirurgicale emportant le trajet de la ponction. L'hyper-éosinophilie capricieuse n'est pas spécifique dans cette localisation mammaire. La sérologie hydatique est d'une grande valeur diagnostique [6]. Très souvent, les circonstances de découverte de l'hydatidose mammaire sont per opératoire ou après l'examen anatomopathologique [7], comme c'est le cas de notre cas. Le traitement curatif de l'hydatidose mammaire est chirurgical. Elle consiste en une kystectomie en bloc, sans le rompre. Dans le cas de son effraction, il faut imbiber le champ opératoire avec du sérum hypertonique ou avec de l'eau oxygénée [3]. Il est important de réaliser une radiographie pulmonaire et une échographie abdominale à la recherche d'autres localisations notamment hépatique et pulmonaire [6]. Dans notre cas, la radiographie thoracique et l’échographie abdominale étaient normales. L’évolution est favorable si l'exérèse chirurgicale est complète, tel est le cas de notre observation.

## Conclusion

L'hydatidose est une maladie ubiquitaire, pouvant atteindre tous les organes. La localisation mammaire en est exceptionnelle, même dans les pays endémiques. Le diagnostic peut être évoqué devant une masse kystique du sein avec des aspects très évocateurs à l'imagerie. Autrement, le diagnostic n'est rapporté qu'après une cytoponction ou une chirurgie d'exérèse qui réalise le traitement de cette pathologique. Le bilan d'extension hydatique doit être réalisé pour différencier entre une localisation primitive ou secondaire de l’échinococcose.
